# Sugar Metabolism Regulates Flavor Preferences and Portal Glucose Sensing

**DOI:** 10.3389/fnint.2018.00057

**Published:** 2018-11-21

**Authors:** Lingli Zhang, Wenfei Han, Chenguanlu Lin, Fei Li, Ivan E. de Araujo

**Affiliations:** ^1^Shanghai Children’s Medical Center, Shanghai Jiao Tong University School of Medicine, Shanghai, China; ^2^Ministry of Education-Shanghai Key Laboratory of Children’s Environmental Health, Xinhua Hospital, Shanghai Jiao Tong University School of Medicine, Shanghai, China; ^3^The John B. Pierce Laboratory, Yale University, New Haven, CT, United States; ^4^Department of Psychiatry, Yale University School of Medicine, New Haven, CT, United States; ^5^Department of Neuroscience, Icahn School of Medicine at Mount Sinai, New York, NY, United States; ^6^Shanghai Engineering Research Center of Tooth Restoration and Regeneration, School & Hospital of Stomatology, Tongji University, Shanghai, China; ^7^Developmental and Behavioral Pediatric Department & Child Primary Care Department, Xinhua Hospital, Shanghai Jiao Tong University School of Medicine, Shanghai, China; ^8^Diabetes, Obesity and Metabolism Institute, Icahn School of Medicine at Mount Sinai, New York, NY, United States; ^9^Department of Cellular and Molecular Physiology, Yale University School of Medicine, New Haven, CT, United States

**Keywords:** dopamine, flavor preferences, glucose metabolism, portal vein, striatum, sugar

## Abstract

In most species, including humans, food preference is primarily controlled by nutrient value. In particular, glucose-containing sugars exert exquisitely strong effects on food choice via gut-generated signals. However, the identity of the visceral signals underlying glucose’s rewarding effects remains uncertain. In particular, it is unknown whether sugar metabolism mediates the formation of preferences for glucose-containing sugars. Using the mouse as a model organism, we made use of a combination of conditioning schedules, gastrointestinal nutrient administration, and chromatographic/electrochemical methods to assess the behavioral and neural effects of activating the gut with either metabolizable glucose or a non-metabolizable glucose analog. We show that mice display much superior preferences for flavors associated with intra-gastric infusions of glucose compared to flavors associated with intra-gastric infusions of the non-metabolizable glucose analog α-methyl-D-glucopyranoside (“MDG,” an activator of intestinal sodium/glucose co-transporters). These effects were unaffected by surgical bypassing of the duodenum, suggesting glucose-specific post-absorptive sensing mechanisms. Consistently, intra-portal infusions of glucose, but not of MDG, induced significant rises in dopamine (DA) levels within brain reward circuits. Our data reveal that the unmatched rewarding effects of glucose-containing sugars cannot be accounted for by metabolism-independent activation of sodium/glucose cotransporters; rather, they point to glucose metabolism as the physiological mechanism underlying the potent reward value of sugar-sweetened flavored beverages. In particular, no circulating “gut factors” need to be invoked to explain the reward value of ingested glucose. Thus, instead of circulating gut hormones, portal-mesenteric sensing of glucose emerges as the preferential physiological pathway for sugar reward.

## Introduction

Sugar-sweetened beverages have been the focus of much attention, as they constitute the largest source of calories and added sugars for both children and adults in the United States (Hu, 2013; Malik et al., 2013; National Cancer Institute, 2013). Accordingly, a large number of epidemiological studies report strong associations between sugar-sweetened beverage intake and weight gain, obesity, type 2 diabetes and coronary heart disease (Johnson et al., 2009; Malik et al., 2013). This is so even when considering the availability of non-caloric alternatives; indeed, the consumption of non-caloric sweeteners remains well below established acceptable daily intake levels (Fitch and Keim, 2012). Notwithstanding the relevance of the issue, the physiological mechanisms mediating this persistent intake of sugar-sweetened beverages remain unclear.

Pairing gut infusions of several macronutrients to the oral ingestion of a distinct flavor results in enduring preferences for that particular flavor, as shown by “flavor-nutrient conditioning” paradigms (Holman, 1968; Sclafani, 2001). However, accumulating evidence reveals that gut infusions of glucose-containing sugars condition flavor preferences with unparalleled strength (Ackroff et al., 2010). Unfortunately, the identity of the physiological signals mediating the unmatched reinforcing strength of glucose remains elusive. In fact, recent findings have shown that intra-gastric infusions of the non-metabolizable glucose analog α-methyl-D-glucopyranoside (“MDG”) are sufficient to condition flavor preferences in mice compared to intra-gastric infusions of non-nutritive solutions (Zukerman et al., 2013). Because MDG acts as an activator of intestinal sodium glucose co-transporters (“SGLTs,” Wright et al., 2011), the Zukerman et al. (2013) findings led to the hypothesis that SGLT activation may be the critical event driving glucose-induced flavor preferences. However, it remains unresolved whether glucose is more reinforcing than its non-metabolizable analogs when compared directly; specifically, it remains undetermined if glucose ingestion generates reinforcement effects that cannot be accounted for by SGLT activation alone.

The goal of the present study is twofold: we tested whether (i) glucose and MDG produce equivalent flavor preferences when compared directly; and (ii) glucose and MDG produce comparable dopamine (DA) effluxes in striatum, a neurochemical event known to mediate sugar reinforcement (Han et al., 2016; Tellez et al., 2016). Based on our previous studies revealing a critical role for glucose metabolism in sugar reinforcement (Ren et al., 2010; Tellez et al., 2013), we predicted that the behavioral and dopaminergic effects of glucose are not explained by intestinal SGLT activation alone.

## Materials and Methods

### Subjects

Forty wild-type adult male mice on a C57BL6/J background were used. At the time of experiments animals were 8–16 weeks old. All experiments were conducted at the J.B. Pierce Laboratory. All experiments were conducted in accordance to NIH rules as well as the J.B. Pierce Laboratory and Yale University regulations on usage of animals in research. The procedures herein performed were assessed and approved by the Pierce/Yale Institutional Animal Care and Use Committee, under protocol IA1-2013.

### Surgical Procedures for Portal-Mesenteric Venous Catheters, Duodenal-Jejunal Bypass and Microdialysis Guiding Cannulae

Once animals had been anesthetized with an intraperitoneal injection of a ketamine/xylazine (100/15 mg/Kg), a midline incision was made into the abdomen. The stomach was exteriorized through the midline incision and a purse string suture was placed into the stomach, into which the tip of MicroRenathane tubing (MRE033, Braintree Scientific Inc., Braintree, MA, USA) was inserted. The purse string was tightened around the tubing, which was then tunneled subcutaneously to the dorsum via a small hole made into the abdominal muscle; a small incision to the dorsum between the shoulder plates was then made to allow for catheter exteriorization. Incisions were sutured and thoroughly disinfected and the exterior end of the catheter plugged.

The same gastric catheter implantation procedure was used in mice sustaining a duodenal-jejunal bypass (DJB) intervention. For these DJB mice, to divert food from its natural course through the duodenum, the latter was separated from pylorus and ligated; the upper jejunum was dissected at 6–8 mm below pylorus, with its distal end end-to-end anastomosed to pylorus, and proximal end end-to-side anastomosed to the lower jejunum 12–14 mm below pylorus, resulting in the bypassing of 6–8 cm of duodenal tissue while preserving pylorus (see Figures 3A–C). For intra-gastric catheters, patency was confirmed by infusing 0.1 mL saline infusions on a daily basis including immediately after the surgeries.

**Figure 1:**
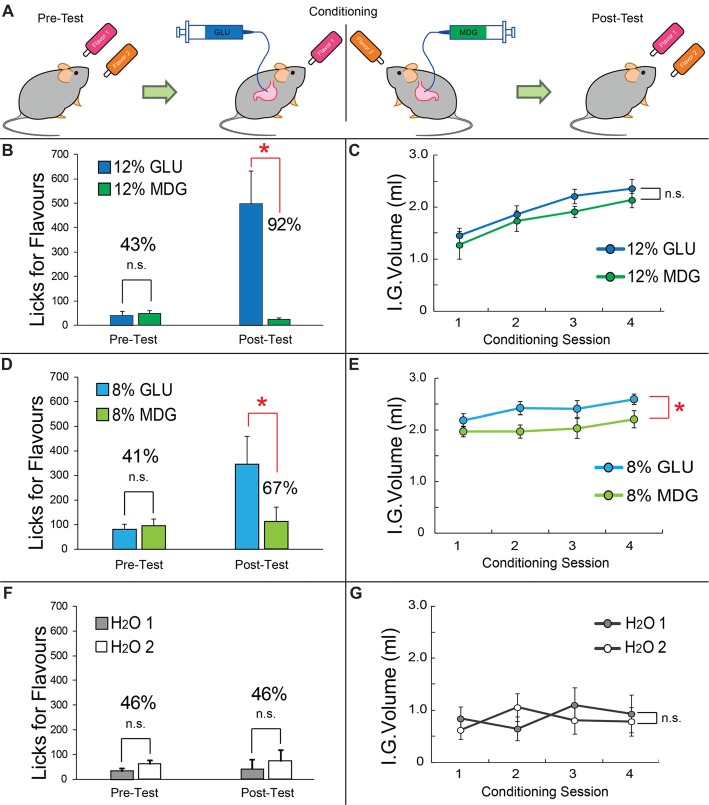
Flavors associated with gut infusions of glucose are significantly preferred over Flavors associated with gut infusions of MDG. **(A)** Flavor-nutrient conditioning paradigm. Previous to the Flavor conditioning sessions, mice were tested for their preferences for the two Flavors in short-term, two-bottle 5 min preference tests in the absence of gut infusions. For condition training, in separate, alternate days, one of the two Flavors (e.g., red) is associated with intra-gastric infusions of glucose (GLU) whereas the second Flavor (e.g., orange) is associated with intra-gastric infusions of the non-metabolizable glucose analog MDG. GLU and MDG solutions were prepared at 12% (because this concentration has been previously shown to induce similar Flavor preferences conditioned by both GLU and MDG (against water infusions) in C57BL6 mice (Zukerman et al., 2013)), 8% and 0%. These “conditioning sessions” lasted for 1 h and were performed for eight consecutive days under food (16 h) deprivation, alternating daily the Flavor-sugar association. Therefore, each Flavor-sugar pair was associated with four conditioning sessions. Importantly, for each animal, Flavors were arbitrarily paired with either GLU or MDG in a balanced design, thereby preventing Flavor identity to influence preference formation. Formation of Flavor preferences were assessed with post-conditioning short-term, two-bottle 5 min preference tests, also in the absence of gut infusions. **(B)** Short-term two-bottle preference tests performed before (left) conditioning sessions reveal no differences between number of licks produced for the 12% GLU-associated vs. 12% MDG-associated Flavor (*post hoc* paired *t*-test, *N* = 6, *p* = 0.36), resulting in a preference of 43% for the 12% GLU-associated Flavor. However, similar tests performed after conditioning sessions (right) show a striking increase in the number of licks for the 12% GLU-associated Flavor (*p = 0.01), resulting in a preference of 92% for the 12% GLU-associated Flavor. **(C)** Number of gut infusions produced throughout the conditioning sessions revealed no major differences between infused volumes of each sugar (two-way ANOVA conditioning session × sugar, *p* = 0.85). **(D)** Short-term two-bottle preference tests performed before (left) conditioning sessions reveal no differences between number of licks produced for the 8% GLU-associated vs. 8% MDG-associated flavor (*post hoc* paired *t*-test, *N* = 6, *p* = 0.2), resulting in preferences of 41% for the 8% GLU-associated flavor. However, after conditioning sessions (right), a significant increase in the number of licks for the 8% GLU-associated flavor (*p = 0.048) was observed, resulting in a preference of 67% for the 8% GLU-associated flavor. **(E)** Number of gut infusions produced throughout the conditioning sessions revealed 8% GLU was infused at higher volumes (sugar effect, *F*_(1,5)_ = 6.7, *p = 0.049). **(F)** A control group was exposed to the exact same protocol as above (panels **B,C**) except that this time licks for either Flavor resulted in gut infusions of water throughout conditioning sessions. Short-term two-bottle preference tests performed before (left) conditioning sessions reveal no differences between number of licks produced for the GLU-associated vs. MDG-associated Flavor (*post hoc* paired *t-test*, *N* = 6, *p* = 0.54), resulting in a preference of 46% for the GLU-associated Flavor. Similar tests performed after conditioning sessions (right) failed to show any increases in the number of licks for the GLU-associated Flavor (*p* = 0.36), resulting in a preference of 46% for the GLU-associated Flavor. **(G)** Number of gut infusions produced throughout the conditioning sessions revealed no differences between infused water volumes associated with each Flavor (two-way ANOVA conditioning session × sugar, *p* = 0.06). GLU, glucose; MDG, α-methyl-D-glucopyranoside; IG, Intra-Gastric infusion; ns, non-statistically significant. Data depicted as mean ± SEM, across animals in all figures.

**Figure 2:**
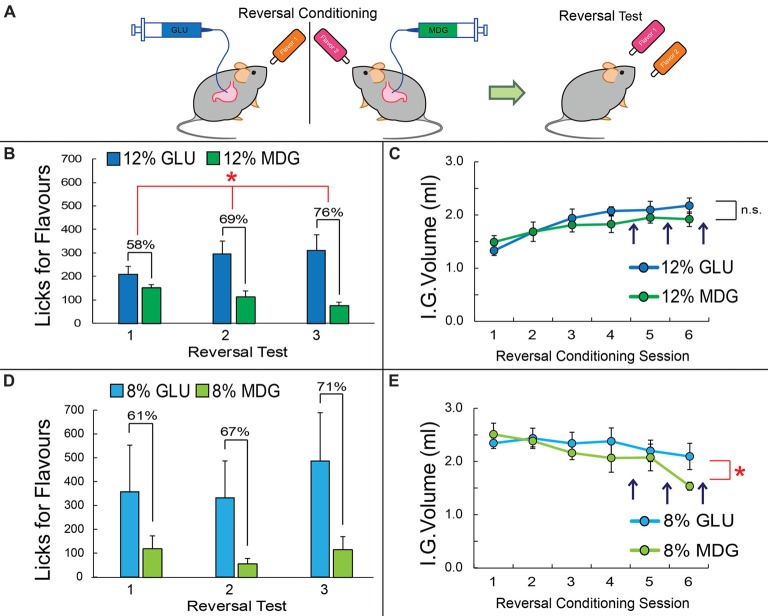
Preferences for glucose-associated flavors are resistant to changes in contingencies. **(A)** After the post-conditioning test, mice previously exposed to the GLU vs. MDG conditioning (shown in Figure 1A) were subjected to reversal learning, that is, the contingencies associating a given Flavor to one of the sugars were arbitrarily reversed (compare against Flavor colors in Figure 1A). **(B)** After four sessions of reversal conditioning, preferences for the now 12% GLU-associated Flavor, i.e., for the Flavor initially associated with MDG, increased from 8% to 58%; a second preference test was performed after five reversal conditioning sessions, which yielded a preference of 69% for the newly GLU-associated Flavor. A third and final preference test was performed after six reversal conditioning sessions, yielding a preference of 78% for the newly 12% GLU-associated Flavor. (Main effect of sugar on licks for Flavors Two-way RM-ANOVA, *p < 0.05). **(C)** Throughout reversal learning, the number of infusions for the 12% GLU vs. 12% MDG did not change significantly across time (two-way RM-ANOVA, *p* = 0.17). **(D)** After four sessions of reversal conditioning, preferences for the 8% GLU-associated flavor increased from 33% to 61%; a second preference test was performed after five reversal conditioning sessions, which yielded a preference of 67% for the newly GLU-associated flavor. A third and final preference test was performed after six reversal conditioning sessions, yielding a preference of 71% for the newly 8% GLU-associated flavor. However, no clear preferences for one flavor vs. another were observed (main effect of sugar on licks two-way RM-ANOVA, *p* = 0.3). **(E)** Throughout reversal learning, the number of infusions for the 8% GLU was significantly higher than 8% MDG with time (two-way ANOVA conditioning session × sugar, *F*_(5,25)_ = 2.7, *p = 0.043). Vertical dark arrows indicate when preference tests were performed. IG, Intra-Gastric infusion; ns, non-statistically significant. Data depicted as mean ± SEM, across animals in all figures.

**Figure 3:**
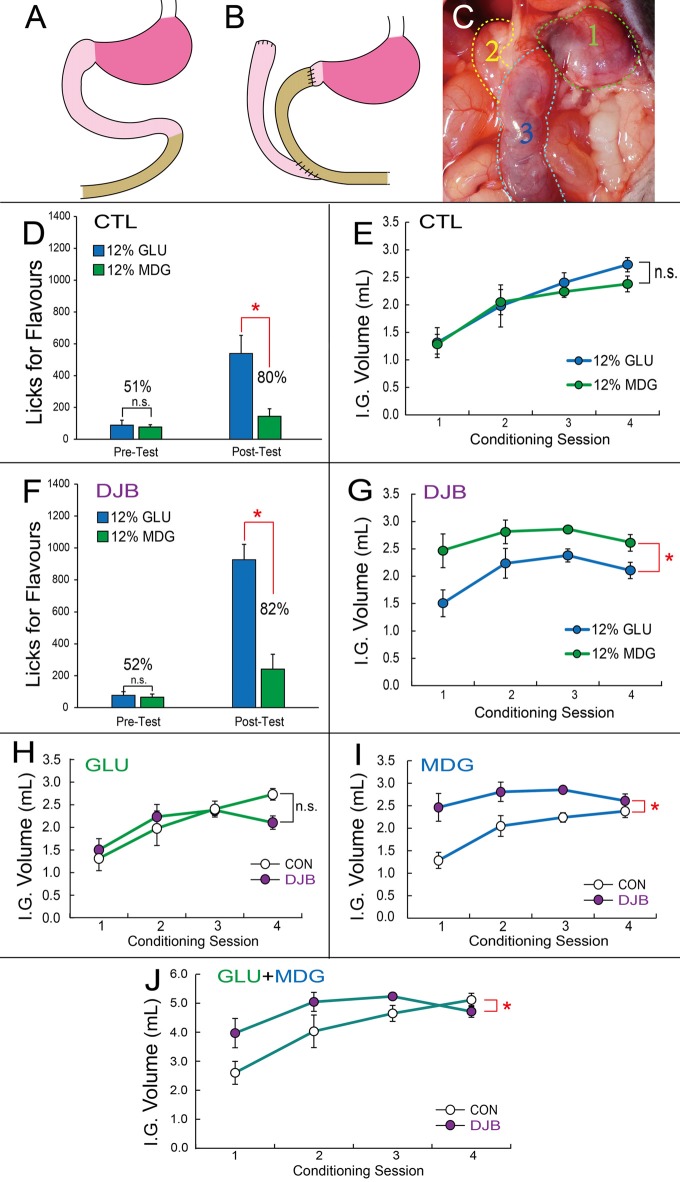
Duodenum jejunum bypass (DJB) mice displayed greater preferences for flavors associated with metabolizable glucose, despite showing decreased satiety for non-metabolizable glucose. **(A,B)** Schematic representation of the DJB intervention, which includes duodenal segmentectomy, jejunal-pylorus end-to-end anastomose and duodenal-jejunal end-to-side anastomose.** (C)** A blue dye was infused through a gastric catheter for assessment of surgical efficacy. In DJB mice the dye was visible only in stomach and jejunum. Legend: (1) Stomach; (2) Duodenum; (3) Distal jejunum. **(D)** In the sham control group, short-term two-bottle preference tests performed before conditioning sessions (left bars) reveal no differences between number of licks produced for the 12% GLU-associated vs. 12% MDG-associated flavor (*post hoc* paired *t*-test, *N* = 5, *p* = 0.8), resulting in preferences of 51% for the 12% GLU-associated flavor. However, after conditioning sessions (right bars), a significant increase in the number of licks for the 12% GLU-associated flavor (*p = 0.016) was observed, resulting in a preference of 80% for the 12% GLU-associated flavor. **(E)** Number of gut infusions produced throughout the conditioning sessions revealed no major differences between infused volumes of each sugar (two-way ANOVA conditioning session × sugar, *p* = 0.49). **(F)** In the DJB group no differences between number of licks produced for the 12% GLU-associated vs. 12% MDG-associated flavor were observed before conditioning sessions (left bars, *post hoc* paired *t*-test, *N* = 5, *p* = 0.7), resulting in preferences of 52% for the 12% GLU-associated flavor. However, after conditioning sessions (right bars), a significant increase in the number of licks for the 12% GLU-associated flavor (*p = 0.004) was observed, resulting in a preference of 82%. **(G)** In the DJB group, number of gut infusions produced throughout the conditioning sessions revealed that 12% MDG was infused at higher volumes than glucose (sugar effect, *F*_(1,5)_ = 13.7, *p = 0.014). **(H)** DJB and control groups self-infused similar amounts of 12% GLU throughout the conditioning sessions (two-way RM-ANOVA *p* = 0.067). **(I)** In the DJB group, number of gut infusions for MDG produced throughout the conditioning sessions was greater than in the control group (two-way ANOVA conditioning session × sugar, *F*_(3,30)_ = 6.887, *p = 0.001). **(J)** The total volume of 12% GLU and 12% MDG gut infusions produced throughout the conditioning sessions revealed overall higher volumes in DJB compared to control mice (two-way ANOVA conditioning session × sugar, *F*_(3,30)_ = 3.921, *p = 0.018). IG, Intra-Gastric infusion; ns, non-statistically significant. Data depicted as mean ± SEM across animals in all figures.

**Figure 4:**
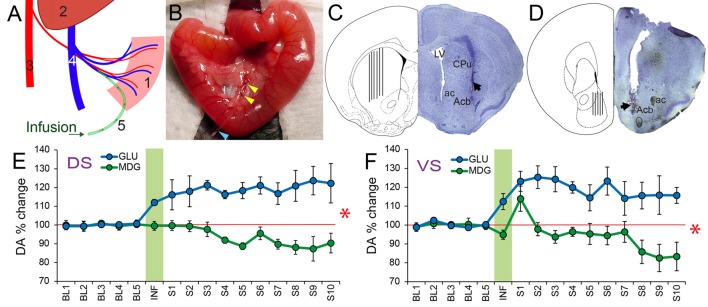
Extracellular levels of DA in Ventral and Dorsal Striatum increase significantly in response to portal infusions of glucose, but not of MDG. **(A)** Schematic representation of the portal catheterization. Legend: (1) Intestine; (2) Liver; (3) Abdominal Aorta; (4) Portal Vein; (5) Infusion catheter.** (B)** Portal catheter inserted into mesenteric vein. Yellow arrowheads indicate the fixation of the catheter; Blue arrowhead points to the catheter.** (C)** The figure shows a coronal section of the mouse brain through the dorsal striatum (caudate/putamen) region. On the right hemisphere is shown a representative Nissl-stained section revealing the tract associated with the tip of an inserted probe (dark arrow). On the left hemisphere is shown the corresponding region in a stereotaxic atlas (Paxinos and Franklin, 2001) containing a depiction of the spread of the actual probe positions. ac, anterior commissure; Acb, Nucleus accumbens of the ventral striatum; CPu, caudate/putamen of the dorsal striatum; LV, Lateral ventricle. **(D)** The figure shows a coronal section of the mouse brain through the Nucleus Accumbens of the ventral striatum. On the right hemisphere is shown a representative Nissl-stained section revealing the tract associated with the tip of an inserted probe (dark arrow). On the left hemisphere is shown the spread of the actual probe positions. ac, anterior commissure; Acb, Nucleus accumbens of the ventral striatum. **(E)** Mice implanted with portal intravenous catheters through which one of glucose or MDG was infused (see Table 1 for parameters). Microdialysis samples were then collected from dorsal striatum in freely behaving animals. Data correspond to timecourse of intestinal sugar effects on DA release (mean ± SEM) of percent DA change with respect to baseline (“BL”) pre-infusion period (Baseline = 100%, red horizontal trace). Vertical green area represents the onset and offset of infusions so that samples collected during this period are named “INF”. “S1–S10” = Samples post-infusion. All samples collected every 6 min. Portal infusions of GLU produced significant increases over baseline, whereas MDG infusions did not (*N* = 5; Two-way RM-ANOVA, stimulus × sample time effect *F*_(15,60)_ = 7.8, *p < 0.001). **(F)** Portal GLU produced significantly greater DA release in ventral striatal compared to MDG (*N* = 5; Two-way RM-ANOVA, stimulus × sample time effect *F*_(15,60)_ = 2.9, *p = 0.001). DA, Dopamine.

For animals used in microdialysis experiments, animals were anesthetized as above. The jejunum was exteriorized through a midline incision. The tip of MicroRenathane tubing (MRE010, Braintree Scientific Inc., Braintree, MA, USA) pre-filled with heparinized saline (100 U/mL), was inserted into the portal-mesenteric vein. For targeting the dorsal striatal region, the animal was placed on a stereotaxic apparatus (David Kopf) under constant flow of ~1% isoflurane anesthesia (1.5 L/min) and a circular craniotomy was drilled at AP = 1.0 mm and ML = ±1.8 mm, to implant guide cannulae (DV = −2.0 mm from skull surface) for posterior insertion of a 2 mm-long microdialysis probe. For targeting the ventral striatal region, a circular craniotomy was drilled at AP = 1.6 mm and ML = ±0.3 mm, to implant guide cannulae (DV = −4.0 mm from skull surface) for posterior insertion of a 1 mm-long microdialysis probe (see Figures 4A–D).

### Stimuli and Behavioral Apparatus

Glucose stimuli (D-Glucose, MDG) were obtained from Sigma (St. Louis, MO, USA) and prepared daily in distilled water at room temperature. For preparing Flavor stimuli, we mixed ~2.0 g of the “4C Totally Light Drink Mix” (wild berry or fruit punch Flavors) powder into 200 mL of a pre-prepared 1 mM sucralose solution. Behavioral experiments were conducted in either one of three identical mouse behavior chambers enclosed in a ventilated and sound-attenuating cubicle (Med Associates Inc., St. Albans, VT, USA). Each chamber is equipped with two slots for sipper tubing placements, at symmetrical locations on one of the cage walls. All sippers are connected to a contact-based licking detection device allowing for measurements of licking responses with 10 ms resolution. All lick timestamps were saved in a computer file for posterior analysis.

### Flavor-Nutrient Conditioning

Mice were trained to produce licks to spouts containing flavored solutions in order to receive intra-gastric infusions of either glucose or MDG depending on the identity of the flavor being ingested. Both solutions were prepared at 8% and 12% because these concentrations have been shown to induce flavor preferences conditioned by either intra-gastric glucose or intra-gastric MDG (compared to intra-gastric water) in C57BL6/J mice (Zukerman et al., 2013). We noted that gut infusions of doses of MDG ≥20% produced clear signs of gastrointestinal discomfort. In DJB and corresponding control mice, only the 12% concentration was used. Plain water was used for control infusions. The exterior part of the gastric catheter was connected to a segment of MicroRenathane tubing secured to the tip of a 3 mL standard syringe containing the solutions to be infused and mounted on the syringe pump. The syringe pump was placed near a small hole made on the superior part of the sound attenuating box in such a way that mice could move freely inside the behavioral chambers. During the task, a detected lick triggered an intra-gastric infusion of the solution that lasted for 3 s at a rate of 0.6 mL/min; therefore, each lick triggered a 30 μL infusion into the stomach. However, licks detected while an infusion was taking place had no programmed consequences (i.e., did not result in additional infusions). These “conditioning sessions” lasted for 1 h and were performed for eight consecutive days under food (16 h) deprivation, alternating daily the flavor-sugar association. Thus, there were four sessions associated with each specific Flavor-sugar pair. Importantly, for each animal, Flavors were arbitrarily paired with either glucose or MDG, thereby preventing Flavor identity to influence preference formation. The same mice were next exposed to a reversal learning protocol, which was identical to the above except that the flavor-sugar associations had been switched.

### Short-Term Two-Bottle Flavor Preference Tests

Short-term (5 min) two-bottle preference tests between the two distinct Flavors were performed previous to and following the conditioning sessions. These tests were performed in extinction, i.e., in the absence of intra-gastric infusions. Short-duration of this test aims to minimize postingestive influences. After conditioning, an identical test was performed to assess the formation of flavor preferences. The number of licks for each Flavor was recorded and used to calculate the preference ratio as follows:

Preference ratio for Flavor1= n(Flavor1)n(Flavor1)+n(Flavor2)

where *n(Flavorx)* denotes the detected number of licks to Flavor *x* during a given session. To eliminate the influence of side-biases mice were tested two consecutive times with sipper positions being switched daily, and the average between the two trials taken as the actual preference. Overall experimental design of the behavioral test is shown in Figure 1.

### Dopamine Measurements During Portal Venous Infusions

Previous to, during, and after portal venous infusions, microdialysate samples from mildly food-deprived awake mice freely moving in their home cages were collected, separated and quantified by HPLC coupled to electro-chemical detection methods (“HPLC-ECD”). Briefly, after a 7 days-long recovery period from surgery, a microdialysis probe (2 mm/1 mm CMA-7, cut off 6 kDa, CMA Microdialysis, Stockholm, Sweden) was inserted into the dorsal/ventral striatum through the guide cannula (the corresponding CMA-7 model). After insertion, probes were connected to a syringe pump and perfused at 1.2 μl/min with artificial CSF (Harvard Apparatus). After a 30 min washout period and a subsequent 30 min pre-intake baseline sampling, dialysate samples were collected every 6 min and immediately manually injected into an HTEC-500 HPLC unit (Eicom, Japan). This sampling resolution match the onset times associated with the ability of adult mice to sense the rewarding properties of sugars independently of sweet taste (~10 min, de Araujo et al., 2008). Analytes were then separated via an affinity column (PP-ODS, Eicom), and compounds subjected to redox reactions within an electro-chemical detection unit (amperometric DC mode, applied potential range from 0 mV to ~2,000 mV, 1 mV steps). Resulting chromatograms were analyzed using the software EPC-300 (Eicom, Japan), and actual sample concentrations were computed based on peak areas obtained from a 0.5 pg/μl DA standard solution (Sigma) and expressed as % changes with respect to the mean DA concentration associated with the baseline (i.e., pre-infusions) sampling period. Locations of microdialysis probes were confirmed histologically.

Doses and infusion parameters were chosen to maximize experimental output yet avoiding over signs of discomfort in animals. All experiments were performed on animals clearly alert and moving naturally in their home cages. Details on each condition are shown on Table 1 below.

**Table 1:** Parameter for sugar infusion during microdialysis sampling.

Infusate	Infusion site	Concentration	Dosage (mg/kg)	Infusion rate (μl/min)	Infusion length (min)
GLU	Portal vein	10%	100	5	6
MDG	Portal vein	10%	100	5	6

*Both GLU and MDG solution used in portal vein infusion were prepared with heparinized saline solution (heparin: 100U/ml). To verify patency of intra-portal catheters, the tubing was filled with 100U/mL heparin and flushed daily until the experiments were performed*.

### Statistical Analyses

Behavioral and neurochemical data analyses were performed using SPSS (PASW Statistics Release 18.0.0) and made use of two- and one-way repeated measures ANOVAs followed by Bonferroni-corrected *post hoc* pairwise comparisons when appropriate. Normality was assessed using SPSS by inspecting and confirming data linearity upon plotting quantile-quantile graphs for every dataset. Violations of sphericity were tested for and ruled out in SPSS by performing Mauchly’s Test of Sphericity for every dataset. Data are reported as mean ± SEM.

## Results

### Flavors Associated With Gut Infusions of Glucose Are Significantly Preferred Over Flavors Associated With Gut Infusions of MDG

During the eight daily Flavor-nutrient conditioning sessions, mice licked spouts containing flavored solutions in order to receive intra-gastric infusions of either glucose (“GLU”) or the non-metabolizable glucose analog “MDG” depending on the identity of the flavor being ingested. Therefore, there were four conditioning sessions associated with each flavor-sugar pair. A depiction of our flavor-nutrient experimental design is shown in Figure 1A.

Pre-conditioning two-bottle short-term tests revealed, as expected, no marked differences in the number of licks produced to each flavor, i.e., previous to either of them becoming associated with gut infusions of GLU or MDG (preference for the GLU-associated Flavor 43%); however, a striking increase in the number of licks for the 12% GLU-associated flavor became evident post-conditioning, to the extent that the preference for the GLU-associated Flavor reached 92% post-conditioning (*N* = 6; two-way repeated measures RM-ANOVA pre- vs. post-conditioning × sugar, *F*_(1,5)_ = 12.3, *p* = 0.01; *post hoc* *t*-test pre-conditioning GLU vs. MDG *t*_(5)_ = 0.9, *p* = 0.3; post-conditioning, *t*_(5)_ = 3.4, *p* = 0.02, see Figure 1B).

Interestingly, analysis of the number of gut infusions produced throughout the conditioning sessions revealed no major differences between the infused volumes of each sugar (*N* = 6; two-way RM-ANOVA conditioning session × sugar, *F*_(3,15)_ = 0.2, *p* = 0.8; see Figure 1C). This rules out potentially aversive MDG effects. The finding is consistent with a previous report where GLU and MDG were shown to independently produce flavor preferences when compared to flavors associated with water infusions (Zukerman et al., 2013). Noticeable however were the increases in the number of infusions throughout conditioning sessions independently of sugar identity (session effect *F*_(3,15)_ = 27.3, *p* < 0.001).

The results above attribute greater reinforcing potency to metabolizable glucose. However, previous findings suggest that, at lower (8%) concentrations, GLU and MDG may act comparably in inducing flavor preferences (Zukerman et al., 2013). We tested this directly by performing the same experiments on an additional, naïve group of mice except that 8% MDG and 8% GLU were used as infusates.

Again, no preferences for either the 8% GLU- or 8% MDG-paired flavors were observed during the pre-conditioning two-bottle short-term tests (preference for the GLU-associated flavor 41%). Now, after eight consecutive days of association training, the preference for the 8% GLU-associated flavor reached 67% during post-conditioning two-bottle short-term tests (*N* = 6; two-way ANOVA pre- vs. post-conditioning × sugar, *F*_(1,5)_ = 9.36, *p* = 0.03; *post hoc* *t*-test pre-conditioning GLU vs. MDG *t*_(5)_ = 1.21, *p* = 0.28; post-conditioning, *t*_(5)_ = 2.61, *p* = 0.048; see Figure 1D).

The overall number of 8% GLU gut infusions during the conditioning sessions was higher than 8% MDG (*N* = 6; main effect of sugar infusate *F*_(1,5)_ = 6.703, *p* = 0.049; see Figure 1E). However, there were no variations in the number of infusions throughout conditioning sessions independently of sugar identity (*N* = 6; two-way RM-ANOVA conditioning session × sugar, *F*_(3,15)_ = 0.37, *p* = 0.7; session effect *F*_(3,15)_ = 1.7, *p* = 0.2).

### Flavor Exposure Did Not Influence Preference Formation

To further assure that flavor exposure *per se* did not influence preference formation, a control group was exposed to the exact same protocol as above except that this time licks for either flavor resulted in dummy gut infusions of nutrients, i.e., water was infused into the gut throughout all conditioning sessions. Pre-conditioning two-bottle short-term tests revealed, as expected, no marked differences in the number of licks produced to each flavor (preference for the GLU-associated Flavor, 46%). Unlike the previous group, this time no increases in the number of licks for the GLU-associated Flavor were evident post-conditioning, to the extent that the preference for the GLU-associated Flavor reached only 46% post-conditioning (*N* = 6; two-way RM-ANOVA pre- vs. post-conditioning × dummy sugar, *F*_(1,5)_ = 0.08, *p* = 0.7; *post hoc* *t*-test pre-conditioning dummy GLU vs. dummy MDG *t*_(5)_ = 0.6, *p* = 0.5; post-conditioning, *t*_(5)_ = 0.9, *p* = 0.3; see Figure 1F). Analysis of the number of gut infusions produced during the conditioning sessions revealed as expected no clear differences in infused water volumes between the two conditions (*N* = 6; two-way RM-ANOVA conditioning session × dummy sugar, *F*_(3,15)_ = 3.1, *p* = 0.06; see Figure 1G). Unlike the previous experimental groups, no apparent increases in the number of infusions throughout conditioning was evident (session effect *F*_(3,15)_ = 0.8, *p* = 0.5); in fact, water infusions remained at significantly lower levels than infusions for either sugar (Figures 1C,E vs. Figure 1G).

### Preferences for Glucose-Associated Flavors Are Resistant to Changes in Contingency

To measure the strength with which GLU produces reinforcement effects that are superior to those produced by MDG, immediately after the post-conditioning test, mice previously exposed to the GLU vs. MDG conditioning (shown in Figure 1A) were subjected to reversal learning, that is, the contingencies associating a given flavor to one of the sugars were arbitrarily reversed (Figure 2A). After four sessions of reversal conditioning, preferences for the now 12% GLU-associated flavor, i.e., for the flavor initially associated with 12% MDG, increased from 8% to 58%; a second preference test was performed after five reversal conditioning sessions, which yielded a preference of 69% for the newly 12% GLU-associated flavor. A third and final preference test was performed after six reversal conditioning sessions, yielding a preference of 78% for the newly 12%GLU-associated Flavor (*N* = 6; two-way RM-ANOVA sugar × reversal test, *F*_(2,10)_ = 8.5, *p* = 0.007; sugar main effects *F*_(1,5)_ = 6.7, *p* < 0.05; Figure 2B). Throughout reversal learning, the number of infusions for the newly 12% GLU-associated vs. 12% MDG-associated Flavors did not significantly change across time (*F*_(5,25)_ = 1.7, *p* > 0.1; Figure 2C).

The same reversal tests were performed on the animals exposed to 8% GLU and 8% MDG conditioning sessions. After four sessions of the 8% GLU and 8% MDG reversal conditioning, preferences for the 8% GLU-associated flavor increased from 33% to 61%. A second preference test was performed after five reversal conditioning sessions, which revealed a preference of 67% for the newly 8% GLU-associated flavor. The final preference test, after six reversal conditioning sessions, revealed a preference of 71% for the newly 8% GLU-associated flavor. However direct tests show that animals failed to form significantly higher preferences for one flavor vs. the other after reversal learning based on 8% infusates (*N* = 6; two-way RM-ANOVA sugar × reversal test, *F*_(2,10)_ = 1.1, *p* = 0.36; sugar main effects *F*_(1,5)_ = 4.0, *p* = 0.1; Figure 2D). In contrast, throughout reversal learning, the number of infusions for the newly 8% GLU-associated vs. 8% MDG-associated flavors was significantly higher across time (*N* = 6; two-way RM-ANOVA conditioning session × sugar, *F*_(5,25)_ = 2.7, *p* = 0.04; see Figure 2E).

Overall, we conclude from the behavioral results above that—since MDG has glucose-like affinity to SGLTs—the robust post-ingestive reinforcement effects of glucose is not accounted for entirely by SGLT activation. Instead, the results indicate that the metabolizable properties of glucose play critical roles in sugar reinforcement.

### Duodenum-Jejunal Bypass (DJB) Mice Display Greater Preferences for Glucose- Over MDG-Paired Flavors Despite Altered Satiation for MDG-Paired Flavors

We next enquired whether duodenal SGLT activation was important for the overwhelming preferences for glucose-paired flavors. Accordingly, we performed similar flavor preference tests on mice sustaining a duodenum-jejunal bypass (DJB) procedure (see “Material and Methods” section and Figures 3A–C).

No preferences for either flavor were observed during the pre-conditioning two-bottle short-term tests in either sham control or DJB groups (Sham Control: 51%; DJB: 52%). After eight consecutive days of association training, preferences for the 12% GLU-associated flavor reached ~80% during post-conditioning two-bottle short-term tests in sham controls (*N* = 6; two-way RM-ANOVA pre- vs. post-conditioning × sugar, *F*_(1,5)_ = 9.263, *p* = 0.029; *post hoc* *t-test* pre-conditioning GLU vs. MDG *t*_(5)_ = 0.275, *p* = 0.795; post-conditioning, *t*_(5)_ = 4.256, Bonferroni *p* = 0.016, see Figure 3D). Similarly, preferences reached ~82% during post-conditioning two-bottle short-term tests in DJB mice (*N* = 6; two-way RM-ANOVA pre- vs. post-conditioning × sugar, *F*_(1,5)_ = 23.243, *p* = 0.005; *post hoc* *t*-test pre-conditioning GLU vs. MDG *t*_(5)_ = 0.341, *p* = 0.747; post-conditioning, *t*_(5)_ = 5.624, Bonferroni *p* = 0.004, see Figure 3F).

#### Altered Satiation for Non-metabolizable Sugar After Duodenal Bypass

During conditioning sessions, the overall number of 12% MDG gut infusions was higher than 12% GLU in the DJB group (*N* = 6; main effect of sugar infusate *F*_(1,5)_ = 13.731, *p* = 0.014; see Figure 3G), but not in the sham control group (*N* = 6; main effect of sugar infusate *F*_(1,5)_ = 1.125, *p* = 0.337; see Figure 3E). Similarly to the previous results shown in Figure 1C, the number of infusions increased significantly throughout conditioning sessions for both sugars in the control group (session effect *F*_(3,15)_ = 22.822, *p* < 0.001; see Figure 3E). However, there were no alterations in the number of infusions across conditioning sessions for the DJB group (*N* = 6; two-way RM-ANOVA conditioning session × sugar, *F*_(3,15)_ = 1.101, *p* = 0.379; session effect *F*_(3,15)_ = 3.222, *p* = 0.053; see Figure 3G).

We analyzed these effects for each sugar separately. No significant differences in number of GLU infusions were observed between the two groups (*N* = 6 each group; two-way ANOVA conditioning session × group, *F*_(3,30)_ = 2.65, *p* = 0.067; see Figure 3H). However, the number of MDG infusions was significantly greater in the DJB group compared to sham controls (*N* = 6 each group; two-way ANOVA conditioning session × group, *F*_(3,30)_ = 6.88, *p* = 0.001; see Figure 3I). Finally, by analyzing the total volume of infused sugar at each conditioning session, we observed greater amounts of infusions DJB compared to sham control mice, especially throughout the initial three sessions (*N* = 6 each group; two-way ANOVA conditioning session × group, *F*_(3,30)_ = 3.921; *p = 0.018; see Figure 3J).

### Extracellular Levels of Dopamine in Both Dorsal and Ventral Striatum Increase Significantly in Response to Portal Vein Infusions of Glucose, but Not of MDG

We then tested whether activation of SGLTs would be sufficient to induce DA efflux in striatum, a neurochemical event critical for food reinforcement (Faure et al., 2005; Sotak et al., 2005). DA release and post-synaptic receptor signaling are required for the formation of glucose-induced Flavor preferences in flavor-nutrient conditioning paradigms (Sclafani et al., 2011). We therefore hypothesized based on the Flavor-nutrient conditioning results above that DA efflux in dorsal and ventral striatum would differ upon portal venous infusions of GLU vs. MDG. Mice were implanted with portal venous catheters and microdialysis samples were collected previous to, during and after the portal infusions (Figures 4A–D). In fact, a small amount slow infusion of GLU (10%, total 30 μl, at 5 μl/min) produced a >20% sustained increase in dorsal striatum DA levels, whereas an equivalent infusion of MDG did not cause DA to raise above baseline levels anytime during the entire session (*N* = 5; Two-way RM-ANOVA, sugar × sample time effect *F*_(15,60)_ = 7.816, *p* < 0.001; see Figure 4E). In ventral striatum, GLU produced a >15% sustained increase of DA levels. In contrast, despite a minor peak of DA release over baseline levels, MDG failed to alter neurotransmitter efflux (*N* = 5; Two-way RM-ANOVA, sugar × sample time effect *F*_(15,60)_ = 2.999, *p* = 0.001; see Figure 4F).

## Discussion

Using a flavor-nutrient conditioning paradigm, we have shown that mice strongly prefer flavors associated with gut infusions of glucose over flavors associated with infusions of the non-metabolizable glucose analog “MDG”. Consistently, glucose but not MDG portal vein infusions produced evident increases in DA levels in both dorsal and ventral striatal reward regions. Overall, our results indicate that glucose metabolism influences brain reward circuitries independently of the activation of gut sodium glucose cotransporters (“SGLTs”): glucose and MDG do in fact have comparable affinities for these cotransporters. Thus, our findings actually demonstrate that glucose-containing sugars drive strong flavor preferences via pathways linked to their metabolization.

Although both glucose and MDG have been shown to be sufficient to induce flavor preferences in mice (Zukerman et al., 2013), a direct comparison between the reinforcing properties of these two sugars hadn’t been previously assessed. The Zukerman et al. (2013) study does however suggest that different physiological pathways mediate glucose vs. MDG reinforcement; in fact, while flavor preferences induced by MDG were abolished upon administering the SGLT blocker phlorizin, the authors needed to supplement the phlorizin solution with a GLUT2 inhibitor in order to abolish glucose-induced flavor preferences. Unfortunately, Zukerman et al. (2013) did not assess the effects of GLUT2 inhibitors independently of SGLT blockers. However, because GLUT2 mediates glucose transport from intestine into portal circulation, we predict that GLUT2 blockers alone would have been sufficient to interfere with the ability of glucose to induce flavor preferences.

More generally, the present study is consistent with previous findings indicating a role for glucose metabolism in sugar reinforcement. In fact, in humans, flavor-nutrient conditioning studies reveal that flavor cues conditioned by a glucose polymer (maltodextrin) intake excite the human ventral striatum in response to increases in plasma glucose levels induced by ingesting the flavored maltodextrin load (de Araujo et al., 2013). This is also in agreement with our previous studies in rodents demonstrating that glucose utilization rates control both nutrient choice in tasteless mutant mice (Ren et al., 2010) and relative preferences between sweeteners in wild-type mice (Tellez et al., 2013). It is also intriguing to note that sugar metabolism seems to control nutrient reinforcement in invertebrate species including *Drosophila*, since tasteless mutant flies were shown to develop strong preferences for metabolizable sugars but not for their non-metabolizable analogs (Burke and Waddell, 2011; Dus et al., 2011). It thus appears that the metabolic control of sugar reward is a highly conserved mechanism whose manifestation involves at least flies, rodents and humans.

The observations above bring about the problem of which physiological signaling pathways may link peripheral glucose sensors to dopaminergic neurons. By using a bariatric DJB model (Han et al., 2016) we observed that the much superior preferences for metabolizable over non-metabolizable glucose were maintained. This is consistent with previous studies showing that jejunal infusions of glucose are sufficient to induce normal flavor preferences in rats (Ackroff et al., 2010). Now, because the jejunum, likewise the duodenum, transports ingested sugars directly into the mesenteric-portal system, we predicted greater brain responses to portal infusions of metabolizable over non-metabolizable glucose. In fact, we observed greater release of the reward-mediating monoamine dopamine upon portal infusions of glucose over MDG in both the dorsal and ventral reward striatal regions.

These findings are important to the extent that they unify the physiologies of sugar reinforcement and glucose metabolism: no putative “gut factors” need be invoked to explain central responses to glucose consumption (Bergman et al., 1982). Specifically, our results rule out the need to invoke circulating gastrointestinal hormones as principal peripheral messengers communicating the ingestion of sugar to reward systems independently of the hepatoportal-brain neural axis as previously suggested (Berthoud, 2008).

Therefore, since striatal dopamine signaling is critical for reward learning in general (Palmiter, 2008), and flavor-glucose conditioning in particular (Touzani et al., 2008), our findings suggest a critical role for portal glucose sensing in sugar reward, in detriment to currently unknown circulating gut factors. Note also that, since ventrolateral striatal sectors play a critical role in orofacial movement (Jicha and Salamone, 1991), portal glucose sensing may provide a physiological link between peripheral glucose sensing and the activation of consummatory oromotor programs. Overall, these new functions for glucose portal sensing reflect the presumed critical role of portal-mesenteric sensors in glucose homeostasis (Bohland et al., 2014).

The above obviously does not rule out a role for brain glucosensing in linking glucose ingestion to dopaminergic activity. Earlier studies showed that central inhibition of glucose utilization is sufficient to elicit feeding (Berthoud and Mogenson, 1977) even in the absence of adrenomedullary responses (Granneman and Friedman, 1983). Specifically, hindbrain catecholamine neurons detect glucose deficits (Ritter et al., 1981; Watts and Donovan, 2009) and may influence dopamine cells directly. Potentially important may be the activity of KATP ion channels expressed on neurons sending afferents to midbrain dopamine cells (Kong et al., 2010), not to mention the possibility that *Substantia nigra compacta* cells may sense glucose directly (Levin, 2000). Returning to the recent *Drosophila* findings, it is interesting to note that SGLT-homologs expressed in brain tissue seem to mediate sugar preferences in tasteless flies (Dus et al., 2011); this implies that glucose entry into brain cells is necessary for sugar reinforcement in flies, as much as intestinal glucose absorption seems to be critical for sugar reinforcement in mammals.

In sum, our findings demonstrate that glucose drives strong flavor preferences and reward dopamine circuitry activity via pathways linked to its metabolization. Future studies must determine the relative roles of peripheral and central metabolic sensors in such effect.

## Author Contributions

IA conceived and designed research. LZ, WH and CL conducted research. LZ, WH, CL and FL analyzed the data. IA wrote the article. All authors actively participated in editing and reviewing the manuscript as well as in interpreting all data.

## Conflict of Interest Statement

The authors declare that the research was conducted in the absence of any commercial or financial relationships that could be construed as a potential conflict of interest.
